# Transcriptome analysis of human brain tissue identifies reduced expression of complement complex C1Q Genes in Rett syndrome

**DOI:** 10.1186/s12864-016-2746-7

**Published:** 2016-06-06

**Authors:** Peijie Lin, Laura Nicholls, Hassan Assareh, Zhiming Fang, Timothy G. Amos, Richard J. Edwards, Amelia A. Assareh, Irina Voineagu

**Affiliations:** School of Biotechnology and Biomolecular Sciences, University of New South Wales, Sydney, NSW 2052 Australia; Faculty of Medicine, University of New South Wales, Sydney, NSW 2052 Australia

**Keywords:** Rett Syndrome, MECP2, Transcriptome profiling, Neurogenetics

## Abstract

**Background:**

MECP2, the gene mutated in the majority of Rett syndrome cases, is a transcriptional regulator that can activate or repress transcription. Although the transcription regulatory function of MECP2 has been known for over a decade, it remains unclear how transcriptional dysregulation leads to the neurodevelopmental disorder. Notably, little convergence was previously observed between the genes abnormally expressed in the brain of Rett syndrome mouse models and those identified in human studies.

**Methods:**

Here we carried out a comprehensive transcriptome analysis of human brain tissue from Rett syndrome brain using both RNA-seq and microarrays.

**Results:**

We identified over two hundred differentially expressed genes, and identified the complement C1Q complex genes (C1QA, C1QB and C1QC) as a point of convergence between gene expression changes in human and mouse Rett syndrome brain.

**Conclusions:**

The results of our study support a role for alterations in the expression level of C1Q complex genes in RTT pathogenesis.

**Electronic supplementary material:**

The online version of this article (doi:10.1186/s12864-016-2746-7) contains supplementary material, which is available to authorized users.

## Background

Rett syndrome (RTT) is an X-linked neurodevelopmental disorder primarily affecting girls at a frequency of 1/10,000 live female births. The core manifestations of RTT include intellectual disability, intractable seizures, spasticity and stereotypic hand movements [1]. More than 95 % of classic RTT cases are caused by sporadic mutations in the gene encoding methyl-CpG binding protein 2 (*MECP2*) [2].

**Fig. 1:**
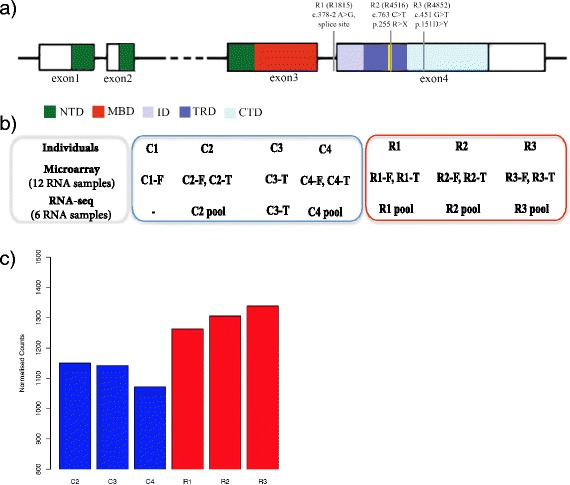
Outline of study design. **a** Localization of MECP2 mutations for the three Rett syndrome patients included in this study. Boxes represent MECP2 exons, and the different colors display distinct protein domains. NTD: N-terminal domain, MBD-methyl binding domain, ID-intervening domain, TRD- transcriptional repression domain, CTD- C-terminal domain. Yellow line- nuclear localization signal. **b** Schematic representation of microarray and RNA-seq data generated for the 3 Rett syndrome cases and 4 controls. F-frontal cortex, T-temporal cortex. **c** *MECP2* expression levels measured by RNA-seq. Y-axis displays normalized RNA-seq counts. No statistically significant difference was observed between Rett syndrome cases and controls

*MECP2* encodes a nuclear protein, which belongs to the methyl-CpG binding protein family and comprises several distinct domains (Fig. 1): a methyl-binding domain (MBD), intervening domain (ID), transcriptional repression domain (TRD), and C-terminal domain (CTD) (Hendrich and Bird, 1998). MECP2 binds with high affinity to mCG dinucleotides, and it has been recently shown to also bind mCA dinucleotides [3]. Although present in most somatic cells, MECP2 expression is most abundant in the brain, with a high ratio of neuronal to glial expression levels [4].

How MECP2 dysfunction leads to the RTT phenotype remains unclear, despite extensive work on multiple mouse models that partially recapitulate the neurological abnormalities of RTT [1]. Gene expression studies of MECP2 transgenic and knockout mice have shown that MECP2 both activates and represses transcription [5, 6] in the mouse brain. While initial studies pointed out to a transcriptional repressor role of MECP2 [7, 8], involving recruitment of NCoR, HDAC3 and Sin3a, more recent data have uncovered an important role of MECP2 as a transcriptional activator [5]. Remarkably, MECP2’s abundance in neuronal nuclei is similar to that of the histone octamer, and thus MECP2 binds globally across the methylated genome [4].

Given the transcriptional regulatory role of MECP2, the underlying mechanism of the RTT phenotype likely results from the dysregulation of MECP2 target genes. Therefore, identifying genes dysregulated in human RTT brain could provide important clues into the mechanism of the disease. However, limited data is available on transcriptome changes in human RTT brain. Previous postmortem brain studies using microarray platforms either failed to identify genes that passed statistical significance criteria after false discovery rate correction or lacked age-matched controls [9–11], and thus little consensus was observed between distinct human studies or between human and mouse data.

Here we carried out the first RNA-seq analysis of brain tissue from RTT patients, and identified several hundreds of differentially expressed genes. We also found a significant overlap between genes downregulated in Rett brain samples and genes activated by MECP2 in mouse models, which included genes involved in the complement cascade.

## Results and discussion

To investigate genome-wide transcriptome changes in human RTT brain, we obtained postmortem tissue from four RTT cases and four age-, sex-, and ethnicity- matched controls (Methods). For each individual brain, we obtained tissue samples from both frontal and temporal cortex (Fig. 1). After quality control assessment of RNA samples two RTT and two control samples were eliminated from further analyses due to low RNA quality (see Methods). The remaining samples, include three Rett cases with the following *MECP2* mutations (Fig. 1a): R1 carries a splice site mutation at the intron3-exon4 junction (c.378-2 A > G), while R2 and R3 carry exon4 mutations: c.763 C > T (p. 255 R > X; transcriptional repression domain) and c.451 G > T (p.151 D > Y; C-terminal domain) respectively. Despite the distinct mutations, the clinical picture was very similar for the three RTT cases. R4852 was diagnosed with RTT with secondary generalised epilepsy and kyphoscoliosis; R4516 was diagnosed with RTT and epilepsy with complex partial seizures; R1815 was diagnosed with RTT, complex partial seizures and scoliosis.

For microarray analyses, each RNA sample was analyzed separately (Fig. 1b, Methods), while for RNA-seq, due to limited amounts of RNA, frontal and temporal samples from the same individual were pooled as shown in Fig. 1b. We obtained an average of 62 million sequencing reads per sample, a high sequencing depth that allowed us to detect lowly expressed transcripts.

**Fig. 2:**
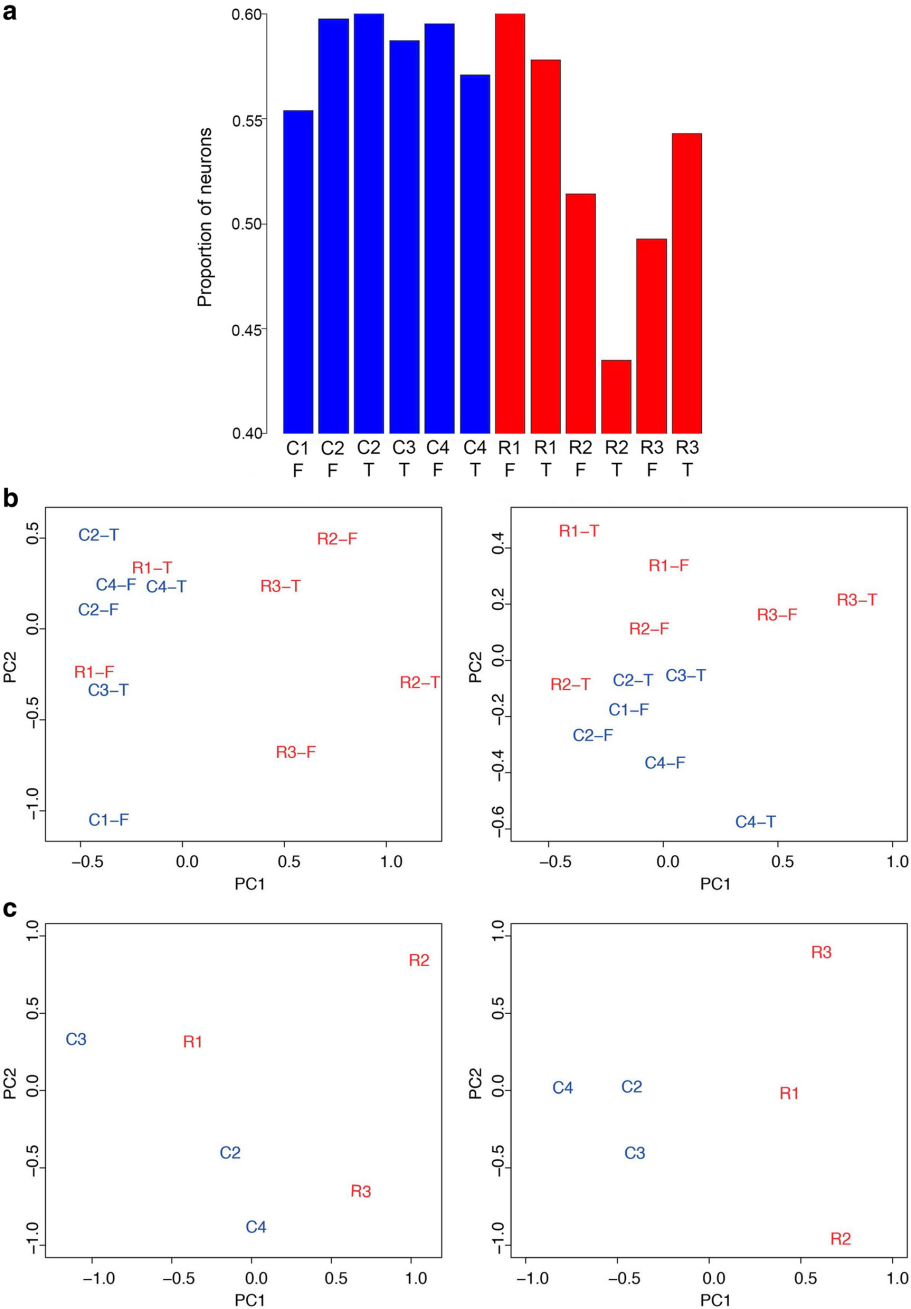
RUV normalization corrects the effects of cellular composition on gene expression data. **a** Proportion of neuronal cells estimated for each sample based on microarray expression data. **b** Principal component plots of microarray expression data before (left) and after (right) RUV normalization. **c** Principal component plots of RNA-seq expression data before (left) and after (right) RUV normalization

Since dissected tissue consists of a mixture of neuronal and glial cells, the results of gene expression analyses could potentially be skewed by differences in cell-type composition across samples. This issue, although important in all transcriptome studies, is particularly important in studies with a small number of samples. However, the potentially confounding effect of cellular composition is rarely addressed due to the difficulty of obtaining good quality RNA from postmortem tissues after laser-microdissection or cell sorting. Thus we used an *in silico* decomposition method [12] to estimate the proportion of neurons and astrocytes in each dissected tissue sample, based on the microarray expression profiles and neuronal and astrocyte cell markers [13] (Methods). We found that although the proportion of neurons showed only minor variations across samples (Fig. 2a, Additional file 1: Table S1, range: 43 % to 60 %, *p* = 0.06, two-tailed *t*-test), the differences in cell type composition affected the clustering of expression profiles (Fig. 2b). The RTT samples that were more similar to controls in terms of cell composition (R1815) clustered with controls, while the rest of RTT samples formed a distinct group (Fig. 2b). To address this issue, we used a normalization method proposed for eliminating unwanted variation, such as experimental co-variates, from transcriptome data (Remove Unwanted Variation (RUV) normalization [14, 15], Methods). RUV estimates a parameter of unwanted variation in the data, i.e. variation not related to the case–control differences, and uses this parameter as covariate in the generalized linear model applied for differential expression (DE) analysis. After applying RUV normalization to both the RNA-seq and microarray data, we found that sample clustering no longer depended on cell-type composition (Fig. 2).

**Fig. 3:**
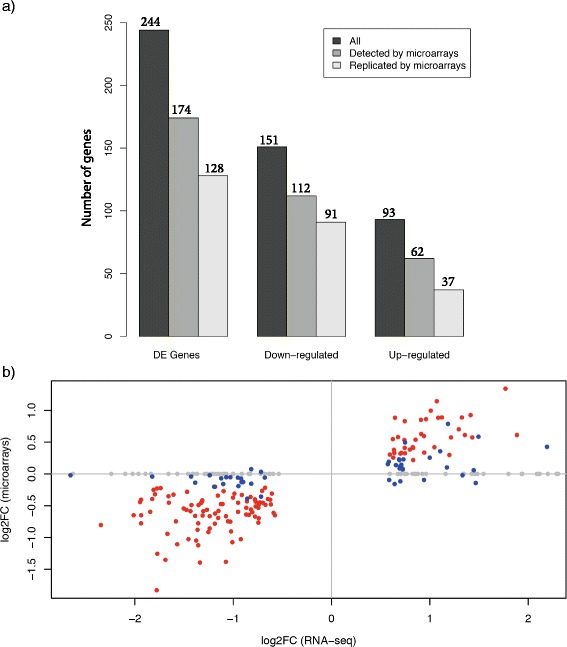
Differentially expression analysis. **a** Number of differentially expressed genes. **b** Scatterplot of log2 fold changes in Rett syndrome samples relative to controls, as detected by RNA-seq (X-axis) and microarrays (Y-axis). All genes identified as differentially expressed genes are displayed, with each gene represented by a dot. Grey dots- genes not detected by microarrays, blue dots- genes that did not reach statistical significance for differential expression based on the microarray data, red dots- genes detected as differentially expressed by both RNA-seq and microarrays

We next analyzed the RNA-seq data to identify genes differentially expressed between RTT samples and controls (Methods). We found that 244 genes (including 14 non-coding RNAs) showed significant expression changes between RTT samples and controls (Fig. 3, FDR < 0.05, Methods), of which 151 were down-regulated and 93 were up-regulated in Rett brain samples.

The use of two independent transcriptome analysis methods (RNA-seq and microarrays), with distinct chemistries, allowed us to assess the technical validation rate of the results on a genome-wide scale, rather than assessing a limited number of genes by a low-throughput method (such as qRT-PCR). Of the 244 differentially expressed genes, 174 were detected on the microarray platform, and 73 % of these were significantly differentially expressed based on the microarray data (FDR < 0.05, see Methods), with the same directionality of expression changes as identified in the RNA-seq data (Additional file 2: Table S2), indicating an appropriate technical validation rate [16, 17].

Given the limited availability of human brain tissue from Rett Synrome patients, we sought to use data from RTT mouse models to assess our results in an independent dataset. To this end, we compared our DE genes with those observed in RTT knock-out and transgenic mice [6]. Ben-Shachar et al.[6] identified genes with consistent expression changes in the cerebellum of MECP2 transgenic and knock-out mice (i.e. significant changes in both mouse models, with opposite directionality). Of the 270 genes identified as activated or repressed by MECP2 in mouse, and passing the detection threshold in our data, 13 genes were also significantly differentially expressed in human RTT samples (Table 1), with the same directionality as in the knock-out mice. These results indicated a significant overlap between the human and mouse differentially expressed genes (*p* = 1.29E-5, hypergeometric test; see Methods), despite the assessment of distinct brain regions. Remarkably, all three genes encoding subunits of the complement C1Q complex (C1QA, C1QB, C1QC) were downregulated in human RTT samples and in MECP2 knock-out mice, and upregulated in MECP2 transgenic mice, strongly suggesting that the expression of these genes is regulated by MECP2.

**Table 1:** Genes differentially expressed in mouse and human Rett syndrome brain

Gene Symbol	FDR	Log2FC	Log2FC	Log2FC
			Mecp2 null/WT	MECP2 Tg/WT
C1QA	0.002	−1.776	−0.278^a^	0.196
CTSS	0.018	−1.407	−0.122^a^	0.291^a^
C1QC	0.030	−1.355	−0.318^a^	0.195
C1QB	0.043	−1.337	−0.287^a^	0.38^a^
B3GNT5	0.019	−1.266	−0.351^a^	0.137
NR4A3	0.003	−0.952	−0.313^a^	0.122
GLRA3	0.041	−0.625	−0.288^a^	0.411^a^
AUTS2	0.027	0.572	0.274^a^	−0.227^a^
FSTL4	0.046	0.723	0.311^a^	−0.117^a^
ESRRG	0.001	0.960	0.226^a^	−0.346^a^
CD83	0.004	1.121	0.096	−0.336^a^
VAV3	0.000	1.326	0.118^a^	−0.313^a^
FKBP5	0.015	1.885	0.087	−0.397^a^

FDR, Log2FC: data from the present study (RNA-seq)

Log2FC Mecp2 null/WT, Log2FC Mecp2 Tg/WT: data From Ben-Shachar et al. [6]

^a^: FDR <0.05

**Fig. 4:**
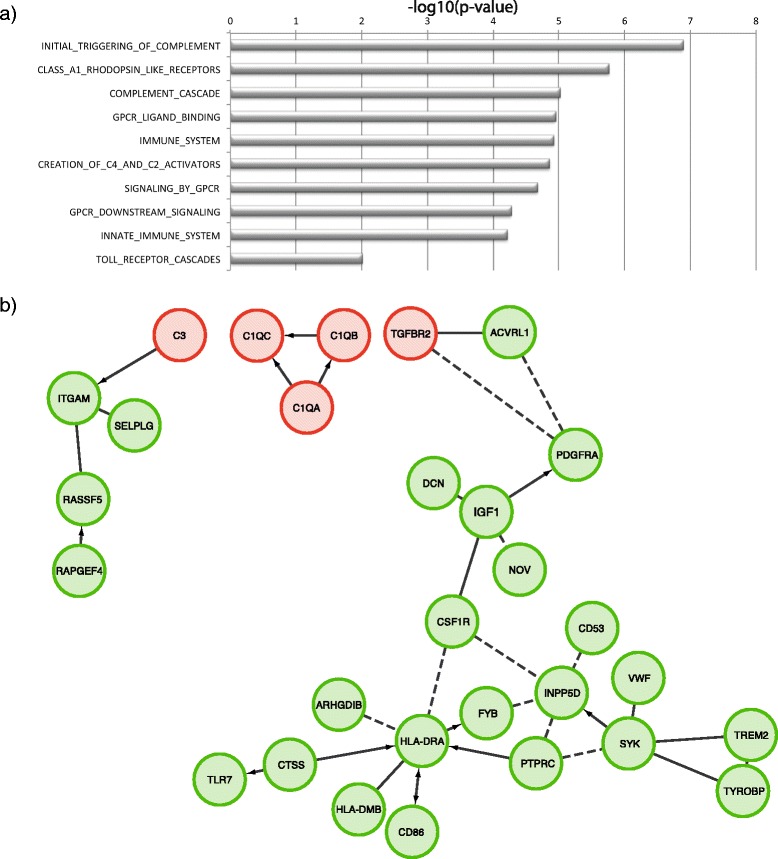
Pathway enrichment analysis of differentially expressed genes. **a** Pathways overrepresented among genes downregulated in Rett syndrome brain. Horizontal bars display the FDR corrected enrichment p-value on a –log10 scale. **b** Schematic display of genes downregulated in Rett syndrome brain (green), which interact with DE genes in the complement pathway (red). Interactions are displayed based on data in the REACTOME database (http://www.reactome.org). The graphical display has been generated using the REACTOME FI plugin for cytoscape

Gene ontology and pathway enrichment analyses (Methods) of down-regulated DE genes showed a significant overrepresentation of genes implicated in immune responses, and the complement cascade in particular (Fig. 4a), while up-regulated genes showed no significant functional enrichment. Notably, in addition to the C1Q complex genes, several other genes that belong to the REACTOME complement cascade pathway (C3, TGFBR2, CX3CR1 and TYROBP) also showed reduced expression in RTT brain (Fig. 4b).

Among the top 10 downregulated genes, two were non-coding RNAs specifically expressed in human brain and testis: RP11-178 F10.3 and RP11-122 F24.1 (-Additional file 3: Figure S1). Their functions are yet uncharacterized, and our data highlights them as relevant for further investigation.

Since a recent study reported that MECP2 primarily represses long genes in the human brain, we assessed the dependency of gene expression changes (log2 fold change) on gene length. We did not observe a significant correlation between gene expression changes and gene length (r2 = -0.02, Additional file 4: Figure S2). Although the top three longest genes were upregulated in RTT brain (Additional file 4: Figure S2), it is difficult to conclude that this observation reflects a general phenomenon of length-dependent transcriptional repression.

We were next interested to know how genes downregulated in RTT brains varied in expression in the normal human brain during fetal and postnatal development. Since the onset of RTT occurs around two years of age, after an initial period of normal development, we hypothesized that MECP2 transcriptional targets might play a particularly important role during this developmental time period.

**Fig. 5:**
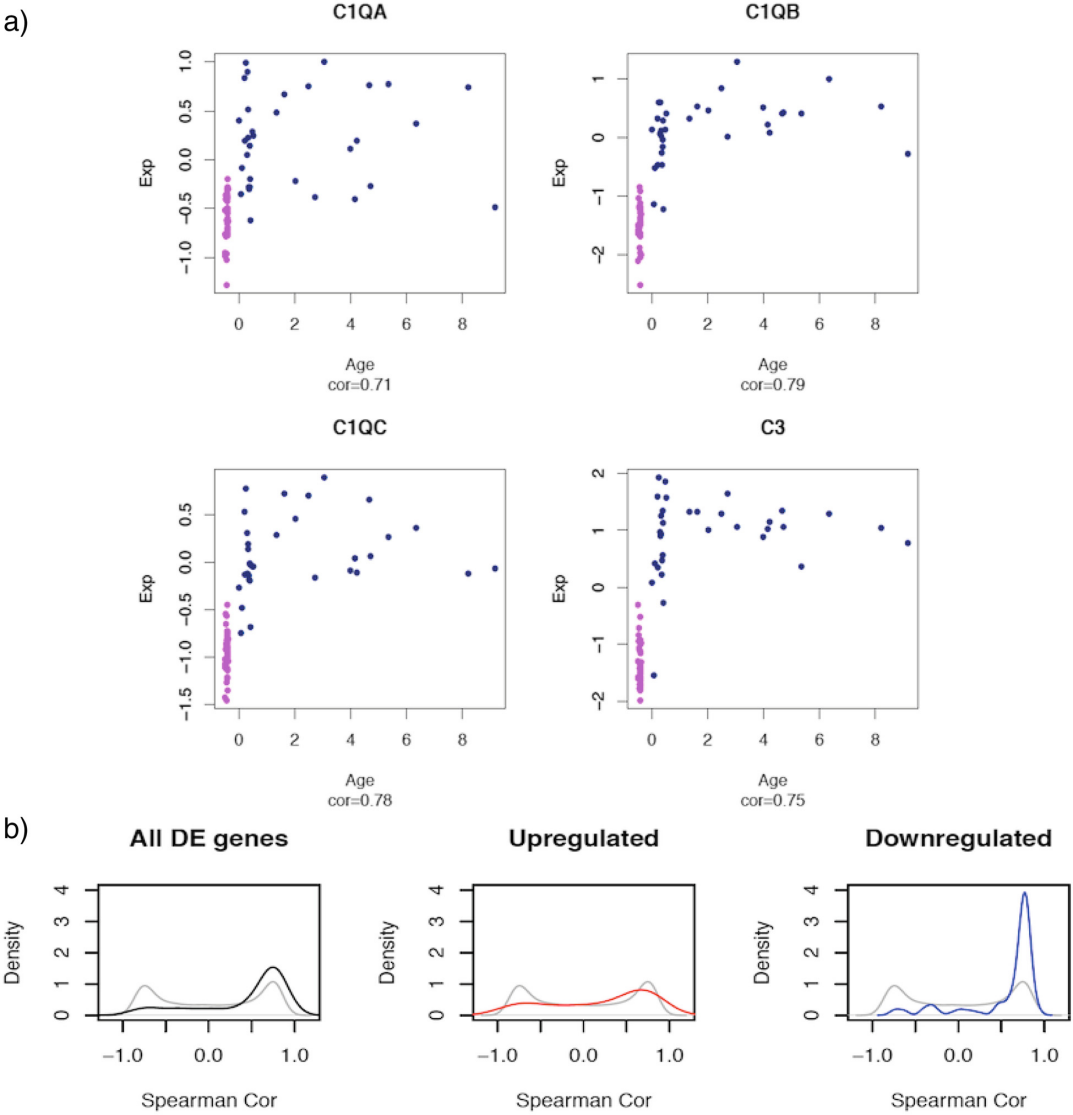
Transcriptional variation of Rett syndrome DE genes in the normal human brain. **a** Scatterplots of gene expression levels (Y-axis) versus age (X-axis). Purple dots: fetal brain samples, dark blue dots: postnatal brain samples. Cor: Spearman correlation coefficients. Data from Colantuoni et al. **b** Density plots of correlation coefficients between expression levels and age. Grey: all genes present in our dataset and the Colantuoni et al. dataset, black: all differentially expressed genes, red: upregulated genes, blue: downregulated genes

To this end, we used transcriptome data from a recent study [18] of gene expression variation in over 200 human prefrontal cortex samples, including 71 samples from fetal and postnatal (<10 years) brains. We assessed the correlation of gene expression with age for all genes detected in our RNA-seq experiment and present in the Colantuoni et al. [18] dataset. As previously reported, the fetal to postnatal transition is associated with major gene expression changes, as most genes undergo either an increase or a decrease in expression during this developmental period. Interestingly, the genes differentially expressed in RTT brain showed primarily an increase in expression levels at the fetal to post-natal transition, a significant shift from the bi-modal distribution of all genes in the dataset (p < 0.05, Kolmogorov Smirnov test). By stratifying the genes into up- and down-regulated, we found that the increase in gene expression at the fetal-to-postnatal transition primarily reflected the behavior of downregulated genes (Fig. 5). Thus genes downregulated in RTT brain increase in expression in normal postnatal brain, suggesting that they may play a functional role during this developmental period.

Our study is the first RNA-seq analysis of post-mortem brain samples from RTT cases and age-, gender- and ethnicity-matched controls. By careful consideration of experimental co-variates such as cellular composition of brain tissue samples, and effective normalization of the data, we were able to detect several hundreds of differentially expressed genes, and observe significant convergence with gene expression changes previously reported in RTT mouse models.

Our data points out a transcriptional deficit of genes encoding components of the C1Q complement complex, as well as several other genes implicated in the complement pathway: C3, TGFBR2, CX3CR1 and TYROBP.

C1Q complex genes are expressed in the brain primarily in microglia, at low levels in resting state, and at higher levels following microglial activation [19]. Although their role has been primarily characterized in neuronflammation, complement proteins play diverse roles in the brain, including a role in synaptic pruning [20]. Interestingly, C1Q genes are expressed in neurons in response to TGFβ receptor (TGFBR2) activation during synaptic pruning [19]. The expression of C1Q genes in response to TGFβ release from astrocytes leads to activation of the complement cascade, and microglial-dependent synaptic pruning [21].

An early transcriptome study of human RTT showed upregulation of glial transcripts and downregulation of neuron-specific mRNAs in post-mortem RTT brain [9]. Whether those results were influenced by the tissue composition of brain samples remains unclear, but our study highlights the importance of considering tissue composition as a covariate in transcriptome analyses of brain tissue samples. Two other studies have previously investigated gene expression changes in RTT genome-wide. Gibson et al [11] compared gene expression in frontal and occipital cortex between six RTT cases and six controls. Due to significant age differences between the Rett and control group, 4–11 years old and 43–52 years old respectively, this study focused on inter-regional differences within each group. Our study on the other hand, was not adequately powered to assess gene expression differences between frontal and temporal cortex for RTT cases and controls separately, and thus larger datasets will be required to further evaluate brain-region specific gene expression differences in RTT.

## Conclusions

Our gene expression data is consistent with one of three possible scenarios (a) a reduction of the total number of microglia in RTT, (b) normal number of resting microglia but reduced microglial activation, (c) reduced expression of C1Q genes in neurons in RTT brain.

It has been previously observed that microglia are depleted in the later developmental stages of RTT mouse brain, after an initial over-activation during early postnatal development [22]. This observation is consistent with our data, showing reduced expression of C1Q complex genes in human RTT brain during adolescence. Whether microglial activation also occurs in the human RTT brain during early postnatal development, is yet unclear. However, reduced dendritic spine densities, which could result from excessive synaptic pruning during early postnatal development, is a feature of RTT documented both in mouse and human brains [23].

Previous mouse studies have highlighted a potential role of astrocytes and microglia in RTT [22, 24, 25]. Restoring MECP2 expression in astrocytes partially restored the neurological phenotypes in a RTT mouse model [25]. In addition, human iPSC-derived astrocytes carrying RTT mutations adversely affect the morphology and function of co-cultured wild-type neurons [24]. Moreover, it has been proposed that repopulating the brain with wild-type microglia by irradiation followed by bone-marrow transplantation could arrest the pathology in a mouse model of RTT [26]. However, the latter result could not be replicated in independent studies [27], indicating that there is yet insufficient evidence to support the use of bone marrow transplantation in RTT patients. Taken together, the results of our study, the first genome-wide assessment of transcriptional changes in human RTT brain, support the notion that alterations in the expression level of C1Q complex genes may contribute to RTT pathogenesis.

## Methods

### Brain tissue samples

Post-mortem brain tissue from four female RTT patients and four age-, sex- and ethnicity- matched controls (Additional file 5: Table S3a) were obtained from the NICHD Brain and Tissue Bank for Developmental Disorders (http://medschool.umaryland.edu/btbank/). All work with these samples was completed in accordance with UNSW ethics requirements for work with human samples (project approval #HC13110). For each brain, approximately 500 mg of frozen tissue was obtained from frontal cortex (BA9 or PFC) and temporal cortex (BA21, BA22, or BA41/42). *MECP2* mutation information was obtained from the NICHD brain bank and confirmed by Sanger sequencing (Additional file 5: Table S3a).

### RNA samples

Total RNA was extracted from around 100 mg of brain tissue using the Qiagen miRNEasy kit following the manufacturer’s protocol, including an on-column DNase I treatment. An additional DNase I (New England Biolabs) treatment was carried out in solution, in order to eliminate any contaminating genomic DNA. Total RNA was eluted in 40 μl RNase-free water and stored at −80 °C. The concentration of RNA and double-stranded DNA (dsDNA) were measured using the Qubit® 2.0 Fluorometer (Life Technologies), and the RNA integrity number (RIN) of each sample was assessed using the Agilent 2100 Bioanalyzer (Agilent Technologies). The RNA and dsDNA concentrations and RIN values are listed in Additional file 5: Table S3b. Only samples with RIN ≥ 5.0 were used in downstream microarray and qRT-PCR analysis. Four RNA samples were excluded from further analysis due to low RNA quality: both RNA samples from the RTT case R4882 (R4882-F and R4882-T), aswell as the control samples C1078-T and C1541-F.

#### Microarray data

Six control RNA samples and six RTT RNA samples (Fig. 1) were analysed on Illumina HumanHT-12 v4 Expression BeadChip arrays. 500 ng of total RNA was processed according to the manufacturer’s protocol and hybridised at the UNSW Ramaciotti Centre for Genomics. All samples were run on the same chip in order to avoid batch effects. Microarray expression data was analysed using the R software (http://www.r-project.org), R package *ruv*13 and the Bioconductor package Lumi [28]. Raw expression data was log_2_ transformed and normalized by quantile normalization. Microarray quality control criteria included high inter-array Pearson correlation coefficients, low variance of mean inter-array correlation and probe detection *P* values <0.05 in at least 30 % of the samples. Further, the ruv package was used to estimate the unwanted variation in the data. As recommended for this method [14], we obtained negative control genes by eliminating the top 5000 genes differentially expressed between RTT samples and controls in a first pass differential expression analysis. We applied the RUV-2 function with default parameters and k = 2, to obtain *w* coefficients, which were then used as covariates in the differential expression (DE) analysis. The choice k = 2 is the minimum k that effectively eliminates the effect of cellular composition on sample clustering. DE was carried out using *limma* [29], followed by Benjamini and Hochberg (BH) correction for multiple comparisons. Genes with FDR-corrected *p*-value < 0.05 were considered differentially expressed.

### RNA-seq data

Strand-specific RNA-seq data was generated from six RNA samples (Fig. 1): a pool of equal volumes of frontal and temporal cortex RNA for each RTT brain (R1815-pool, R4516-pool, R4852-pool) and two of the control brains (C1078-pool, C1571-pool), as well as temporal cortex RNA from a third control sample (C1541-T). 1–5 μg of total RNA was depleted of ribosomal RNA using the Epicentre Ribo-zero kit, according to the manufacturer’s protocol. Library preparation using the Illumina TruSeq Stranded kit (http://www.illumina.com/products/truseq_stranded_total_rna_library_prep_kit.html) and sequencing on an Illumina HiSeq 2500 sequencer were carried out at the UNSW Ramaciotti Centre for Genomics. Libraries were barcoded and sequenced in one lane, thus avoiding lane effects, to obtain 100 bp paired-end reads. An average of 60 million reads were obtained for each sample (Additional file 6: Table S4). Sequencing reads were trimmed to eliminate adaptor sequences using *Trimmomatic* [30] aligned to the Human Genome (hg19) using the TopHat spliced aligner [31] with default parameters. Quality-control assessment of RNA-seq data was carried out using the RNAseqQC software [32]. Gene-level expression quantification was carried out using the Rsubread featureCount function with the following parameters: useMetaFeatures = TRUE, allowMultiOverlap = FALSE, minMQS = 10, requireBothEndsMapped = TRUE, countChimericFragments = FALSE [33]. Genes with expression level >0.5 FPKM in at least 3 of the 6 samples were retained for further analysis.

TMM (Trimmed Mean of M-values) normalised data was analysed for a first pass differential expression analysis using edgeR [34], in order to define negative control genes as described above for microarrays. The unwanted variation coefficients (*w*) were then estimated using the RUVg function [15] with default parameters and k = 1, and further used as covariate in the differential expression analysis. Differential expression analysis was carried out using edgeR with BH correction for multiple comparisons. Genes with FDR-corrected *p*-value < 0.05 were considered differentially expressed. The differential expression analysis results for RNA-seq and microarray data are provided in Additional file 7: Table S5 and Additional file 8: Table S6 respectively.

### Tissue composition analysis

The cellular composition of brain tissue samples was assessed *in silico,* using the *ssKL* algorithm in CellMix R package [12], based on quantile normalised (but not log2 transformed) microarray expression data. Cell-type specific marker gene lists for neurons and astrocytes were obtained from Cahoy et al. [13]. The estimated proportions of astrocytes and neurons in each sample are listed in Additional file 1: Table S1.

**Gene ontology and pathway enrichment analysis** was carried out using the GOseq package [35], with gene-length adjustment for RNA-seq data. Gene ontology terms with BH corrected *p*-value < 0.05 were considered significant. Pathway enrichment analysis was carried out using GSEA (http://www.broadinstitute.org/gsea/index.jsp) and the Reactome pathway database (http://www.reactome.org/). The gene ontology and pathway enrichment results are listed in Additional file 9: Table S7 and Additional file 10: Table S8.

### Comparison with mouse data

The overlap between the 244 genes identified as differentially expressed in the present study (i.e. 1.3 % of the entire dataset) and genes differentially expressed in mouse models of RTT [6] was carried out using a hypergeometric test implemented in the *phyper* function in R. 270 genes had been identified as differentially expressed in *MECP2* knockout and transgenic mice, and were quantified in our dataset, and 13 of those (i.e. 4.8 %) were also identified as dysregulated in human RTT brain, a statistically significant overlap (*p* = 1.29E-5).

## Abbreviations

BA, brain area; BH, Benjamini and Hochberg; CTD, C-terminal domain; DE, differentially expressed; ID, intervening domain; MBD, methyl-binding domain; PFC, prefrontal cortex; RTT, Rett syndrome; TRD, transcriptional repression domain.

## Additional files

Additional file 1: Table S1. Tissue Composition. (XLS 6 kb) Additional file 2: Table S2. List of Differentially Expressed Genes (RNA-seq). (XLS 50 kb) Additional file 3: Figure S1. Expression levels of RP11-178F10.3 and RP11-122F24.1 across human tissues. Boxplots display expression levels based on RNA-seq data from the GTEx Consortium. The plots were generated using the GTEx potal online tool: http://www.gtexportal.org. (JPG 280 kb) Additional file 4: Figure S2. Dependence between gene expression changes and gene length in Rett syndrome brain. Scatterplot displays gene length on the X-axis and fold-changes in Rett syndrome samples relative to control samples, as measured by microarrays, on the Y-axis, for all genes in the dataset. Genes identified as significantly differentially expressed are shown in red, while all other genes are shown in black. (JPG 96 kb) Additional file 5: Table S3. Brain tissue and RNA samples. (XLS 10 kb) Additional file 6: Table S4. RNA-seq data overview. (XLS 7 kb) Additional file 7: Table S5. Differential Expression Results, RNA-seq. (XLS 2488 kb) Additional file 8: Table S6. Differential Expression Results, Microarrays. (XLS 2356 kb) Additional file 9: Table S7. Gene Ontology Enrichment Results for Downregulated Genes. (XLS 68 kb) Additional file 10: Table S8. Pathway Enrichment Results for Downregulated Genes. (XLS 12 kb)
