# Supplementary figures and images for: Transcriptome analysis of human brain tissue identifies reduced expression of complement complex C1Q Genes in Rett syndrome
Source: BMC Genomics. 2016 Jun 6;17:427. doi: 10.1186/s12864-016-2746-7 (PMC4895974; doi:10.1186/s12864-016-2746-7)

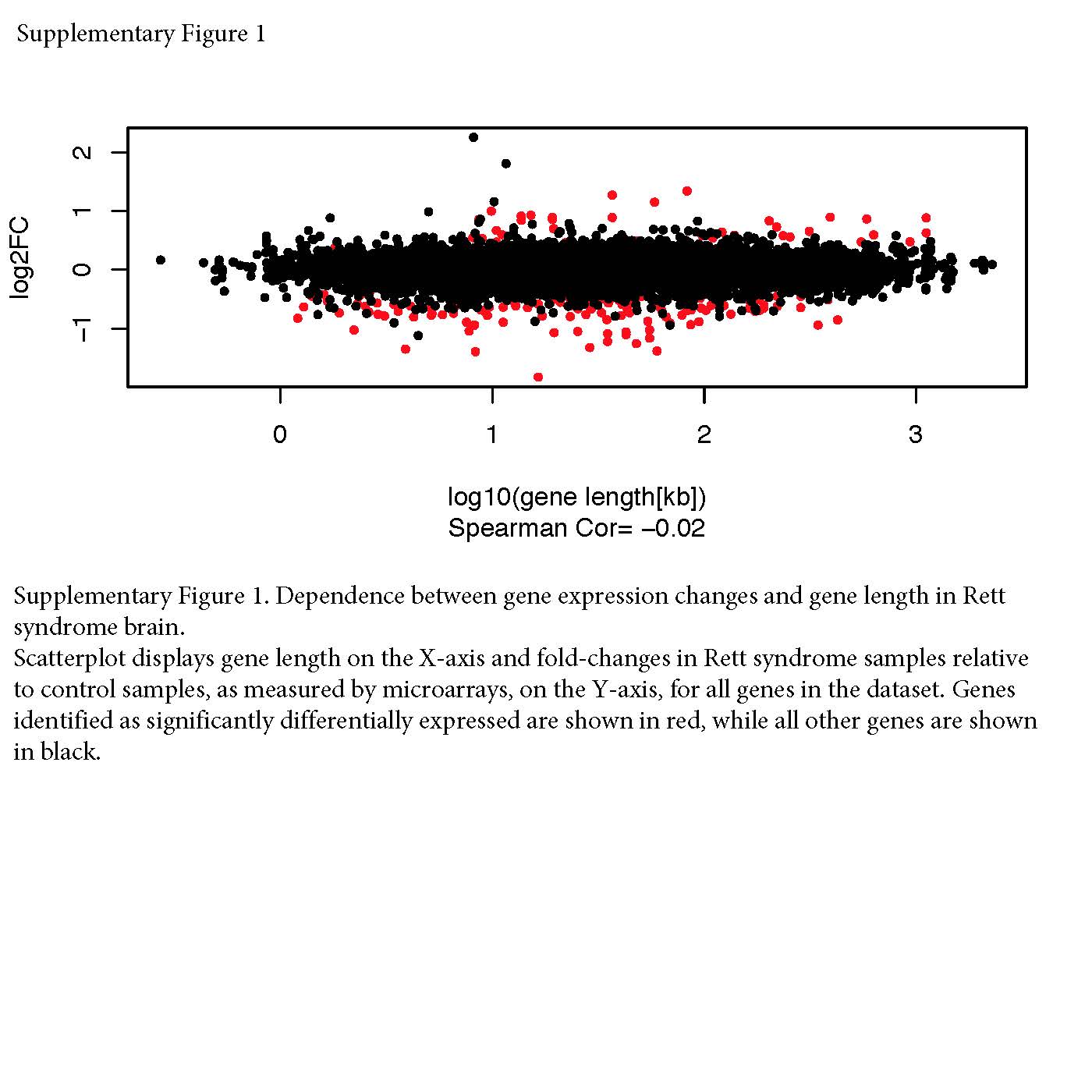

Supplement: Additional file 3: Figure S1. — Expression levels of RP11-178F10.3 and RP11-122F24.1 across human tissues. Boxplots display expression levels based on RNA-seq data from the GTEx Consortium. The plots were generated using the GTEx potal online tool: http://www.gtexportal.org. (JPG 280 kb) [file 12864_2016_2746_MOESM3_ESM.jpg]

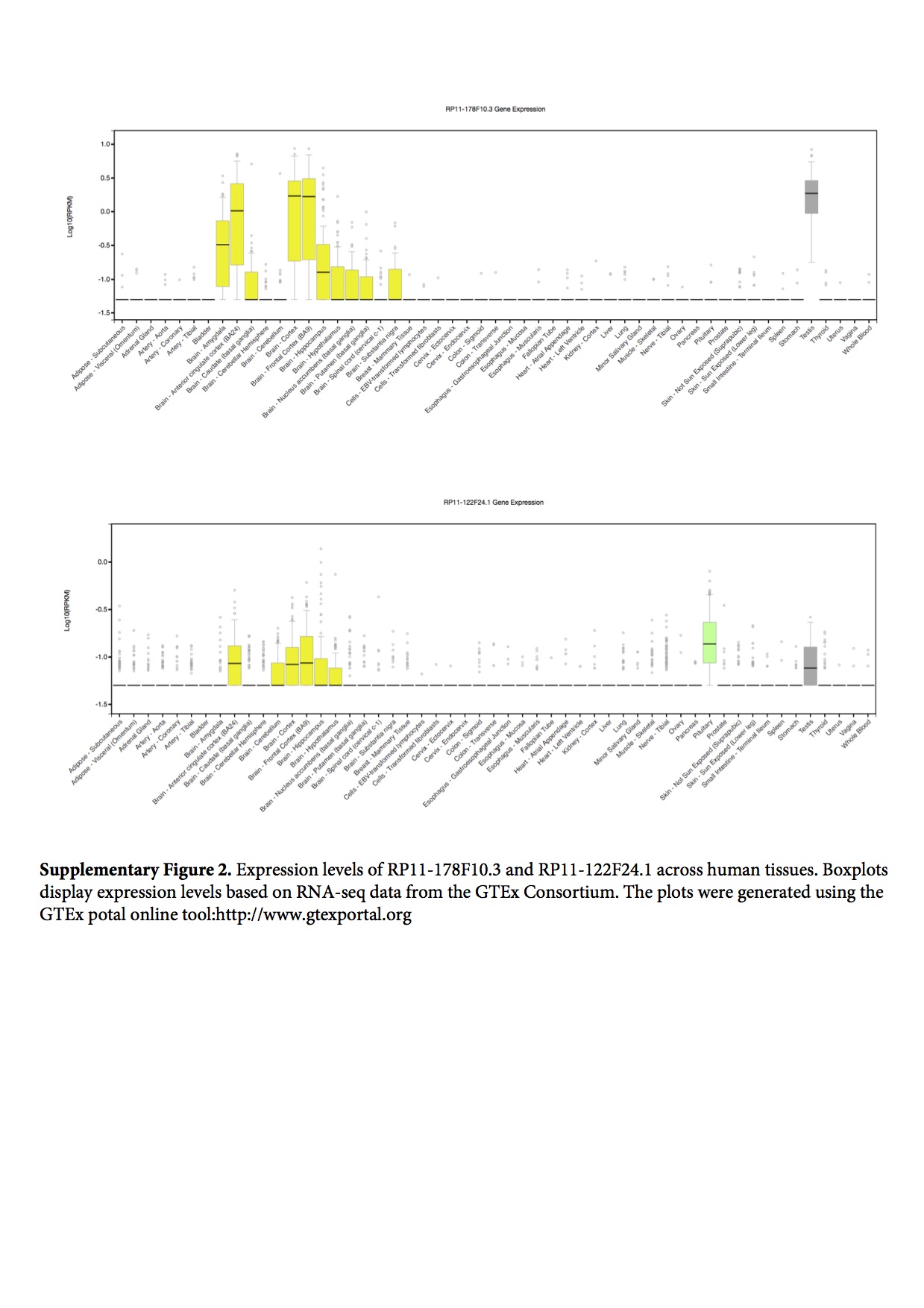

Supplement: Additional file 4: Figure S2. — Dependence between gene expression changes and gene length in Rett syndrome brain. Scatterplot displays gene length on the X-axis and fold-changes in Rett syndrome samples relative to control samples, as measured by microarrays, on the Y-axis, for all genes in the dataset. Genes identified as significantly differentially expressed are shown in red, while all other genes are shown in black. (JPG 96 kb) [file 12864_2016_2746_MOESM4_ESM.jpg]
